# Supplementary material for: Transcriptome Analysis Reveals Vimentin-Induced Disruption of Cell–Cell Associations Augments Breast Cancer Cell Migration
Source: Cells. 2022 Dec 13;11(24):4035. doi: 10.3390/cells11244035 (PMC9776984; doi:10.3390/cells11244035)
Supplement: Supplementary file 1 [file cells-11-04035-s001.zip › DEGs and enrichment analysis.zip!/DEGs and enrichment analysis/WT_VIMvsVector KEGG/UP/WT_VIMvsVector_UP.pathway_map.zip!/WT_VIMvsVector_UP.pathway_map/src/hsa00240.html]

hsa00240


- hsa:1841

- Up regulated genes

ENSG00000168393(1.71585159334733)

- hsa:7083

- Up regulated genes

ENSG00000167900(2.41029212356598)

- hsa:7298

- Up regulated genes

ENSG00000176890(1.69666136230634)

- hsa:1841

- Up regulated genes

ENSG00000168393(1.71585159334733)

- hsa:7083

- Up regulated genes

ENSG00000167900(2.41029212356598)

- hsa:1854

- Up regulated genes

ENSG00000128951(2.04672197440859)

Close
